# Editorial: Methods in experimental pharmacology 2023

**DOI:** 10.3389/fphar.2025.1531464

**Published:** 2025-01-21

**Authors:** Somasundaram Arumugam, Amit Khurana, Kala Kumar Bharani

**Affiliations:** ^1^ Department of Pharmacology and Toxicology, National Institute of Pharmaceutical Education and Research (NIPER)-Kolkata, Kolkata, West Bengal, India; ^2^ Department of Pediatrics, Division of Gastroenterology and Hepatology, School of Medicine, University of California San Diego, La Jolla, CA, United States; ^3^ Department of Veterinary Pharmacology and Toxicology, College of Veterinary Science (CVSc), P.V. Narsimha Rao Telangana Veterinary University (PVNRTVU), Warangal, Telangana, India

**Keywords:** experimental pharmacology, animal model, stomatitis, attention deficit-hyperactivity disorder (ADHD), aging

## Introduction

Experimental pharmacology is a branch of medical science that focuses on studying the effects of drugs through controlled laboratory experiments. It involves *in vitro* (test tube or cell culture), *in vivo* (animal or human) models, *ex vivo* (tissues or organs), *in silico* (computer-based) and genetic/molecular/imaging techniques. Together, these methods enable researchers to understand drug efficacy, safety, and mechanisms before clinical trials. All experiments involving animals must adhere to the 3Rs principle (Replacement, Reduction, Refinement) and require approval from ethical review boards such as the Committee for the Control and Supervision of Experiments on Animals (CCSEA) in India and similar legal organizations upholding the ethical aspects of animal research throughout the world.

This Research Topic aimed to highlight the latest and most innovative experimental techniques and methods used to investigate fundamental pharmacological questions. Various types of papers, such as review articles or opinions on methodologies or applications, including advantages and limitations, were invited with a focus on shedding light on current technologies and up-to-date methods showing promise in helping to advance the field. In this Research Topic, four interesting papers are published, and we will discuss a brief outline of them here.

Vitamia and group designed an interesting two-arm, double-blinded, randomized controlled trial enrolling patients with recurrent aphthous stomatitis (RAS) to investigate the efficacy of an α-mangostin hydrogel film with a chitosan alginate base and was compared with a placebo over 7 days. Currently, they are in the process of recruiting participants for the trial. An earlier study has demonstrated that an *in vivo* investigation of α-mangostin hydrogel films with a chitosan-alginate base for RAS therapy exhibited a favourable healing response, achieving a healing rate of 93% on the seventh day (Milanda et al., 2022). Similarly, hydrogel film dosage form in transdermal delivery offers several advantages, such as extended and targeted active substance release, enhanced bioavailability, improved solubility, cost efficiency and reduced dosing frequency (Yang and Pierstorff, 2012). These promising reports led them to conduct a clinical trial of α-mangostin hydrogel films with a chitosan-alginate base to evaluate the clinical efficacy and safety in treating patients with RAS.

The antifungal drug fluconazole is a third-generation triazole drug widely used to treat systemic and superficial fungal infections (Lee et al., 2021). Irrespective of the broad spectrum of activity and good safety profile, it has limited solubility, requires higher oral doses, and has several negative impacts. Topical dosage forms are available in the market; however, they still face challenges due to limited solubility and skin permeation (Gupta et al., 2010). Cheng et al. have formulated transfersomes of fluconazole to enhance its solubility and, ultimately, antifungal activity. Transfersomes were formulated by a thin-film hydration technique using soya lecithin and Span 80 and evaluated for entrapment efficiency and *in vitro* drug diffusion. They have used various methods to assess the prepared transfersomes, such as scanning electron microscopy, differential scanning calorimetry (DSC), vesicle size, polydispersity index (PDI), zeta potential, and antifungal activity. The *in vitro* and *ex vivo* drug diffusion and antifungal studies confirmed better diffusion and enhanced antifungal potential of fluconazole in transfersomal formulation. The developed transfersomal gel is postulated as a superior alternative for enhanced fluconazole delivery, highlighting its potential for future clinical applications.

Herbal preparation of Long Mu Qing Xin Mixture (LMQXM) is one among various traditional methods to treat attention deficit hyperactivity disorder (ADHD), a prevalent neurodevelopmental disorder in children (Chen and Xiao, 2016; Li et al., 2023). Li et al. have scrutinized the potential pharmacological mechanisms by which LMQXM improves behaviour in spontaneously hypertensive rats (SHR/NCrl). They have reported that LMQXM ameliorated hyperactivity and learning and memory deficits of SHR/NCrl rats in addition to upregulated dopamine, norepinephrine, adenylate cyclase and cAMP levels, and the expression of proteins and genes associated with the DRD1/cAMP/PKA-CREB pathway in prefrontal cortex (PFC) and striatum of the rats. This study tentatively suggests that LMQXM may resolve hyperactivity and learning and memory deficits of SHR/NCrl rats by elevating catecholamine neurotransmitters in the PFC and striatum. This elevation of catecholamine neurotransmitters appears to be linked to the activation of the DRD1/cAMP/PKA-CREB signalling pathway, which is expected to yield potentially optimal results at moderate doses.

Aging is a complex, naturally occurring process resulting in a deterioration in cellular homeostasis and increased vulnerability to stressors (Santamaria-Garcia et al., 2023). Reports indicate metabolic diseases expedite human aging (Tang et al., 2013). Sarkar et al. reviewed the scientific evidence exploring potential mechanisms behind the onset and development of cardiovascular and metabolic Research Topic, particularly exacerbated with aging. The age-associated oxidative load and redox imbalance are contributing factors for cardiometabolic morbidities via vascular remodelling and endothelial damage. Recent evidence has claimed the importance of gut microbiota in maintaining regular metabolic activity, which declines with chronological aging and cardiometabolic comorbidities. Genetic mutations, polymorphic changes, and environmental factors strongly correlate with increased vulnerability to aberrant cardiometabolic changes by affecting key physiological pathways. They have suggested that collaborative efforts between healthcare providers, researchers, and policymakers are crucial in effectively addressing the challenges of the aging population.

## Conclusion

Though this Research Topic has attracted a limited number of papers, experimental pharmacology is an exciting area that provides the scope for drug discovery and evaluation. This process involves preclinical screening before any potential drug candidate proceeds to clinical study. All four papers are very interesting and cover a mixture of four different areas. We believe that this Research Topic has addressed a few critical areas under the category of Experimental Pharmacology, which may inspire the readers for their research.
